# Real world experience of R‐CHOP with or without consolidative radiotherapy vs DA‐EPOCH‐R in the first‐line treatment of primary mediastinal B‐cell lymphoma

**DOI:** 10.1002/cam4.2347

**Published:** 2019-07-02

**Authors:** Esther Hian Li Chan, Liang Piu Koh, Joanne Lee, Sanjay De Mel, Anand Jeyasekharan, Xin Liu, Tiffany Tang, Soon Thye Lim, Miriam Tao, Richard Quek, Mohamad Farid Bin Harunal Ras, Yuh Shan Lee, Colin Diong, Daryl Tan, Seok Jin Kim, Yen Lin Chee, Li Mei Michelle Poon

**Affiliations:** ^1^ Department of Haematology‐Oncology National University Hospital Singapore Singapore; ^2^ Department of Medical Oncology Singapore General Hospital Singapore; ^3^ Department of Haematology Singapore General Hospital Singapore; ^4^ Department of Medicine Samsung Medical Center Sungkyunkwan University School of Medicine Seoul South Korea

**Keywords:** lymphoma

## Abstract

Primary mediastinal large B‐cell lymphoma (PMBCL) is a distinct clinico‐pathological subtype of diffuse large B‐cell lymphoma with unclear prognostic factors and limited clinical data. Optimal treatment and role for radiotherapy is not fully defined. We performed a multicenter retrospective review of 124 patients with newly diagnosed PMBCL between 2001 and 2016. Treatment regimens were R‐CHOP (n = 41), R‐CHOP + RT (n = 37), and DA‐EPOCH‐R (n = 46). 6% (n = 3) in the DA‐EPOCH‐R group received RT. With a median follow up of 45 months, the overall 5‐year OS and PFS was 89.4% and 82.4%, respectively. The type of chemo‐radiotherapy regimen, B symptoms and Ann‐Arbor staging showed a significant association with OS on univariate analysis but only B symptoms remained prognostic (*P* = 0.012) after multivariate analysis. The chemo‐radiotherapy regimen, Japanese IPI and Ann‐Arbor stage was significantly associated with PFS in univariate analysis, but only chemo‐radiotherapy regimen remained significant (*P* = 0.02) after multivariate analysis. Patients who received R‐CHOP + RT or DA‐EPOCH‐R had better PFS than those receiving R‐CHOP alone, with 5‐year PFS of 90% vs 88.5% vs 56%, respectively (*P* = 0.02). In the subgroup analysis of patients with bulk (n = 71), R‐CHOP alone (n = 21) had inferior 5‐year PFS 56.6% compared to those who received R‐CHOP + RT (n = 23) 91.3% or DA‐EPOCH‐R (n = 27) 92.6% (*P* = 0.007). In contrast, in patients without bulk (n = 42), there was no impact of treatment regimen on PFS (*P* = 0.25). In conclusion, R‐CHOP + RT and DA‐EPOCH‐R provide excellent outcomes in patients with PMBCL. In patients with bulky disease, the use of DA‐EPOCH‐R may be preferable as it allows omission of RT without reduction in efficacy.

## 1 INTRODUCTION

Primary mediastinal large B‐cell lymphoma (PMBCL) is an uncommon B‐cell lymphoma accounting for 2%‐4% of all non‐Hodgkin lymphomas. It typically occurs in young females, who present with bulky anterior mediastinal mass causing superior vena cava syndrome. Although PMBCL was previously classified as a subtype of DLBCL, it has specific clinical, histological, and molecular features that distinguish it from DLBCL and overlap instead with nodular‐sclerosis classical Hodgkin lymphoma.^1^ This has led PMBCL to be recognized as a unique entity by the World Health Organization classification of lymphoid tumors since 2001.

The optimal chemotherapy regimen and the role of radiotherapy in PMBCL remains an area of research. Prior to the introduction of rituximab, dose‐dense and dose‐intense second‐ and third‐generation protocols such as VACOP‐B (etoposide, doxorubicin, cyclophosphamide, vincristine, prednisone, and bleomycin) showed better patient outcomes over CHOP (cyclophosphamide, vincristine, doxorubicin, and prednisolone) chemotherapy.^2^, ^3^, ^4^ However, the addition of rituximab to CHOP has significantly improved the event free survival (EFS) and overall survival (OS) in PMBCL and a number of studies have suggested no benefit for dose‐intensified regimens when compared with R‐CHOP ± radiotherapy (RT) in the rituximab era.^5^, ^6^, ^7^, ^8^, ^9^

Despite the good results with R‐CHOP + RT, a number of issues remain. First, about 20%‐30% of patients experience progression or relapses with poor outcomes.^10^ Second, given the younger age and female predominance in PMBCL, significant concerns remain with respect to toxicity from mediastinal RT, particularly the increased risk of secondary breast cancer and cardiotoxicity.^11^, ^12^ Lastly, whether consolidative RT can be omitted using dose‐intensified regimens or in low‐risk patients treated with R‐CHOP remains controversial.

Risk stratification with IPI in PMBCL is of limited value due to the young age and limited stage of PMBCL at presentation. Aoki et al identified a subgroup of patients with favorable prognostic factors (low IPI and without pleural or pericardial effusion) given R‐CHOP without RT with an excellent 4‐year PFS and OS of 87% and 95%, respectively, compared with those with high IPI and pleural or pericardial effusion (4‐year PFS and OS 54% and 81%, respectively).^13^ These findings require further validation but suggest low‐risk patients may be adequately treated with R‐CHOP alone, while high‐risk patients may require a more intensive approach.

In 2013, NCI published the results of a single‐center Phase 2 study with 51 PMBCL patients given a rituximab‐containing dose‐intensive, dose‐adjusted (DA) multi‐agent chemotherapy regimen, DA‐EPOCH‐R (etoposide, prednisone, vincristine, cyclophosphamide, doxorubicin, rituximab) which showed an excellent 5‐year EFS and OS of 93% and 97%, respectively, despite omission of RT in the majority of patients.^14^ However, there are no randomized comparisons of DA‐EPOCH‐R with R‐CHOP in PMBCL. Although results from the randomized CALBG/Alliance 50303 trial comparing DA‐EPOCH‐R with R‐CHOP in large B‐cell lymphoma showed no difference between the two regimens,^15^ PMBCL patients comprised only 5% (N = 28) of all patients and no definitive conclusions are possible. Shah et al reported a large retrospective multicenter comparison of R‐CHOP vs DA‐EPOCH‐R in the front‐line setting.^16^ They reported improved complete remission rates and similar PFS in the DA‐EPOCH‐R arm, despite only 13% of patients in the DA‐EPOCH‐R arm receiving RT, compared to 59% in the R‐CHOP arm. However, there was increased treatment‐related toxicity in patients receiving DA‐EPOCH‐R

The aim of this multicenter study was to retrospectively evaluate the overall clinical outcomes of a large consecutive series of PMBCL patients. We also looked at the impact of treatment regimen (R‐CHOP, R‐CHOP with consolidation RT (R‐CHOP + RT) or DA‐EPOCH‐R) on survival in all patients, and specifically in patients with bulky disease, and the prognostic value of IPI and the Japanese prognostic index on patient outcomes.

## 2 METHODS

### 2.1 Study design and population

All adults (>18 years) with newly diagnosed PMBCL as defined by the World Health Organization 2008 classification were identified from pathology databases in two tertiary hospitals in Singapore and one in South Korea between 2005 and 2016. A total of 124 patients with clinicopathological features consistent with PMBCL, treated with one of three treatment regimens (R‐CHOP, R‐CHOP with consolidation RT (R‐CHOP + RT) or DA‐EPOCH‐R) and sufficient follow‐up data were included. Patients with human immunodeficiency virus positive serology were excluded. The study approved by Institutional Review Boards of participating centers.

### 2.2 Primary treatment regimen

All 124 patients received rituximab‐based treatment. A total of 41 patients received R‐CHOP alone, 37 patients received R‐CHOP + RT, and 46 patients received DA‐EPOCH‐R The R‐CHOP regimen comprised of prednisolone 100 mg D1‐5, rituximab 375 mg/m^2 ^D1, cyclophosphamide 750 mg/m2 D1, doxorubicin 50 mg/m^2^ D1, vincristine 1.4 mg/m^2^ capped at 2 mg D1, while the DA‐EPOCH‐R regimen used was as published in the original paper.^14^ RT was provided at the physician's discretion.

### 2.3 Data collection

Pre‐treatment clinical parameters extracted included age, gender, ECOG performance status, International Prognostic Index (IPI) score, bulk (defined as nodal/mediastinal mass ≥ 10 cm), B symptoms, Ann‐Arbor stage, and extranodal sites. Patients were also scored for the Japanese PMBCL prognostic score, based on presence of IPI ≥ 3 and the presence/absence of pleural or pericardial effusion. Treatment details included the type of first‐line treatment regimen and use of consolidation RT. Therapy response was evaluated by clinical examination and computed tomography scan or fluorodeoxyglucose positron emission tomography (FDG‐PET) imaging node regions according to standardized response criteria for non‐Hodgkin lymphomas/PMBCL.^17^

### 2.4 Statistical analysis

OS was calculated from date of diagnosis until date of last appointment or date of death while PFS was calculated from date of diagnosis until date of relapse, progression or death, whichever occurred first. Survival was assessed by Kaplan‐Meier methods and Cox‐regression modelling was used for univariate and multivariate analyses on SPSS software, Version 13.

## 3 RESULTS

### 3.1 Patient characteristics

A total of 124 patients were included in the analysis (Table 1). The median age at diagnosis was 27 years (range 11‐72 years) and 69 (56%) patients were female. The majority had early stage disease (Stage I‐II, 71%) and 63% of patients had bulky disease. Most patients had a low (IPI 0‐1) (n = 84, 69%) or intermediate risk IPI (IPI 2‐3) (n = 34, 28%). Twenty (19%) of 106 patients with data available had a Japanese prognostic score (either IPI ≥ 3 and the presence/absence of pleural or pericardial effusion) of 1 or 2 at diagnosis.

**Table 1:** Patient characteristics in the different treatment groups

Characteristic	All, N=124 (%)	RCHOP group, N=41 (%)	R‐CHOP+RT group, N=37 (%)	R‐EPOCH group, N=46 (%)
Median age, range (years)	27 (11‐72)	28 (11‐72)	26 (14‐48)	27 (16‐51)
Female gender	69 (56)	21 (51)	22 (60)	26 (56.5%)
Stage (N=123)^a^				
Stage I‐II	87 (71)	23 (57)	32 (86)	32 (70)
Stage III‐IV	36 (29)	17 (43)	5 (14)	14 (30)
B symptoms (N=110 with data)	46 (42)	12 (29)	13 (35)	21 (6)
Bulky disease (N=113)^a^	71 (63)	21 (51)	23 (62)	27(58)
IPI (N=121)^a^				
IPIO‐1	84 (69)	22 (54)	30 (83)	32 (73)
IPI 2‐3	34 (28)	16 (39)	6 (17)	12 (27)
IPI 4‐5	3 (3)	3 (7)	0 (0)	0 (0)
Japanese Score (N=106)^a^				
0	86 (81)	21 (70)	33 (97)	36 (78)
1	14 (13)	6 (20)	2 (3)	7 (15)
2	6 (6)	3 (10)	0 (0)	3 (6)

a Number with data available.

### 3.2 Primary treatment regimen

A total of 41 patients (33%) were treated with R‐CHOP alone, 37 (30%) patients with R‐CHOP ± RT and 46 (37%) with DA‐EPOCH‐R Only three patients (6%) in the DA‐EPOCH‐R group received RT (Table 1). The choice of initial regimen varied according to individual institution practices and year of enrollment. There were no significant differences between the groups in age, sex, or bulky disease (defined as nodal/mediastinal mass ≥ 10cm). There were more patients with advanced stage disease, higher risk IPI or higher risk Japanese scores receiving R‐CHOP compared to the other two regimens.

### 3.3 Clinical outcomes

**Figure 1:**
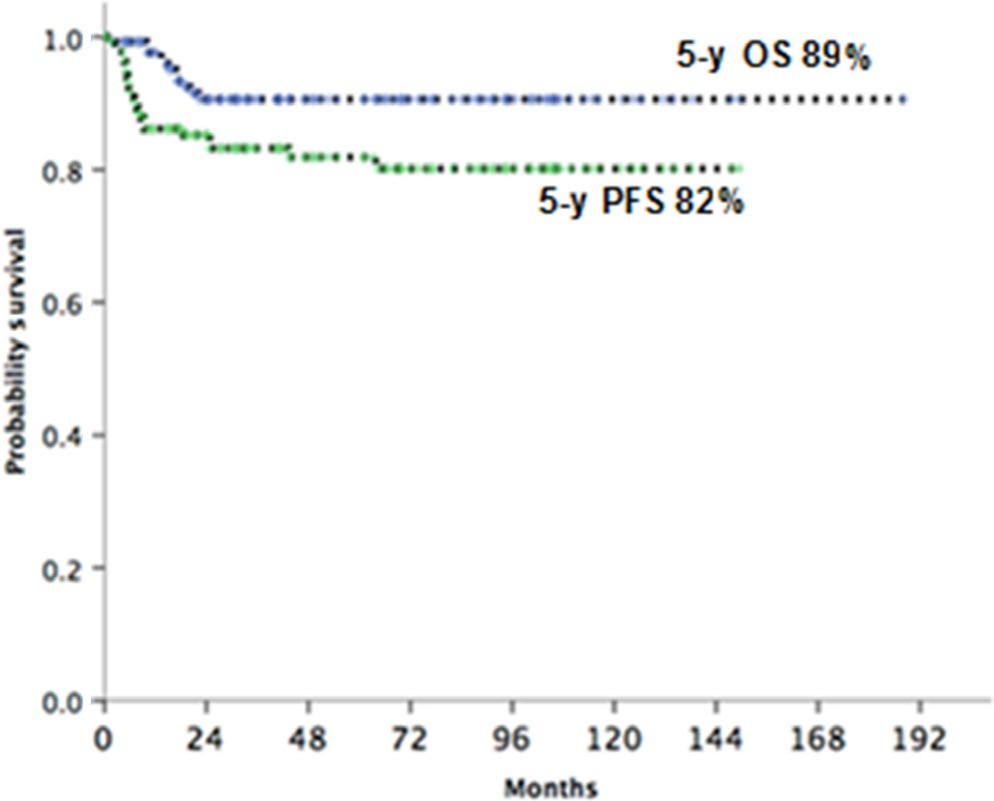
5 y PFS and OS for the whole cohort

The median follow‐up of the entire cohort was 45 months. The 5‐year OS and PFS for the whole cohort was 89.4% (95% CI, 81.6%‐94.0%) and 82.4% (95% CI, 73.7%‐88.5%) respectively (Figure 1). There were no treatment related mortality and toxicities observed were similar to other publications.^16^

### 3.4 Impact of treatment regimen on survival and prognostic factor analysis

The analysis of prognostic factors was done on the following parameters: age, sex, International Prognostic Index (IPI) score, bulk (defined as nodal/mediastinal mass ≥ 10cm), B symptoms, Ann‐Arbor stage, treatment regimen, and Japanese prognostic score for 124 patients with available data (Table 1).

### 3.5 Progression Free Survival (PFS)

**Figure 2:**
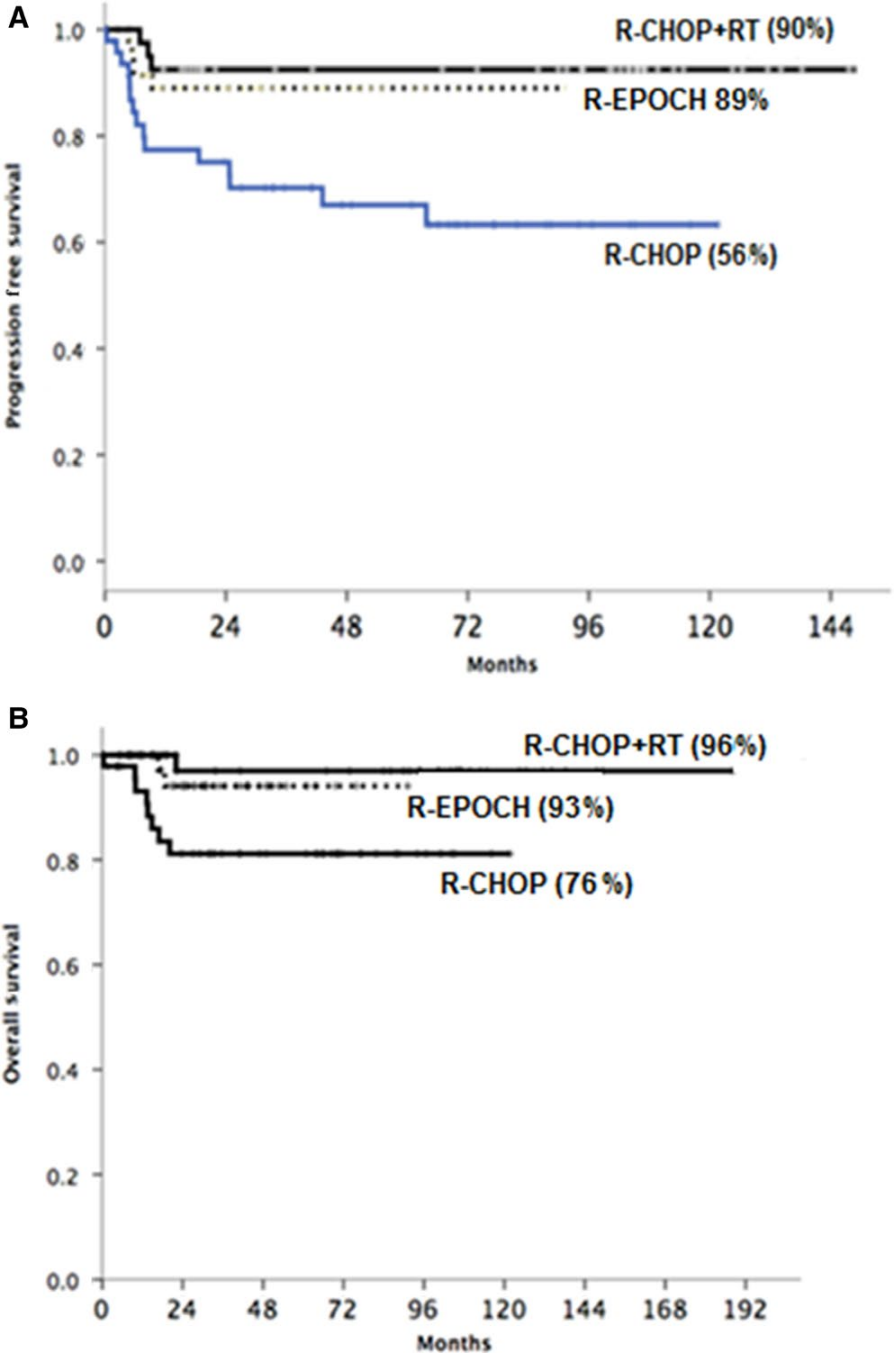
(A) 5 y PFS for R‐CHOP, R‐CHOP + RT and R‐EPOCH (B) 5yr OS for R‐CHOP, R‐CHOP + RT and R‐EPOCH

Univariate analysis identified the treatment regimen (*P* = 0.005), the Japanese prognostic score (*P* = 0.02), and Ann Arbor stage (*P* = 0.005) to be significantly associated with PFS. On multivariate analysis that adjusted for the factors above, only treatment regimen was found to have significant impact on PFS (*P* = 0.02). Patients who received R‐CHOP alone had significantly inferior PFS compared to patients who received R‐CHOP + RT (HR, 0.25; 95% CI, 0.06 to 0.98; *P* = 0.047) or DA‐EPOCH‐R (HR, 0.25; 95% CI, 0.084 to 0.74; *P* = 0.012) with a projected 5‐year PFS of 56.5% (95% CI 33.6%‐74.1%), 88.5% (95% CI 74.5%‐95.1%), and 90% (95% CI 72.1%‐96.7%), for R‐CHOP, DA‐EPOCH‐R, and R‐CHOP + RT, respectively (Figure 2A).

### 3.6 Overall survival (OS)

On univariate analysis, only the treatment regimen (*P* = 0.010), presence of B symptoms (*P* = 0.012), and Ann‐Arbor staging (*P* = 0.021) showed a significant association with OS. The 5‐year OS for patients receiving R‐CHOP, DA‐EPOCH‐R, and R‐CHOP + RT were 76.1% (95% CI, 57.1% to 87.3%), 93.9% (95% CI, 77.8% to 98.4%), and 96.9 (95% CI, 79.8% to 99.5%), respectively (Figure 2B). However, on multivariate analysis, only presence of B symptoms retained prognostic significance (HR 3.27, 95% CI 0.37‐30.01; *P* = 0.012).

### 3.7 Effect of treatment regimen on PFS in patients with bulky disease

**Figure 3:**
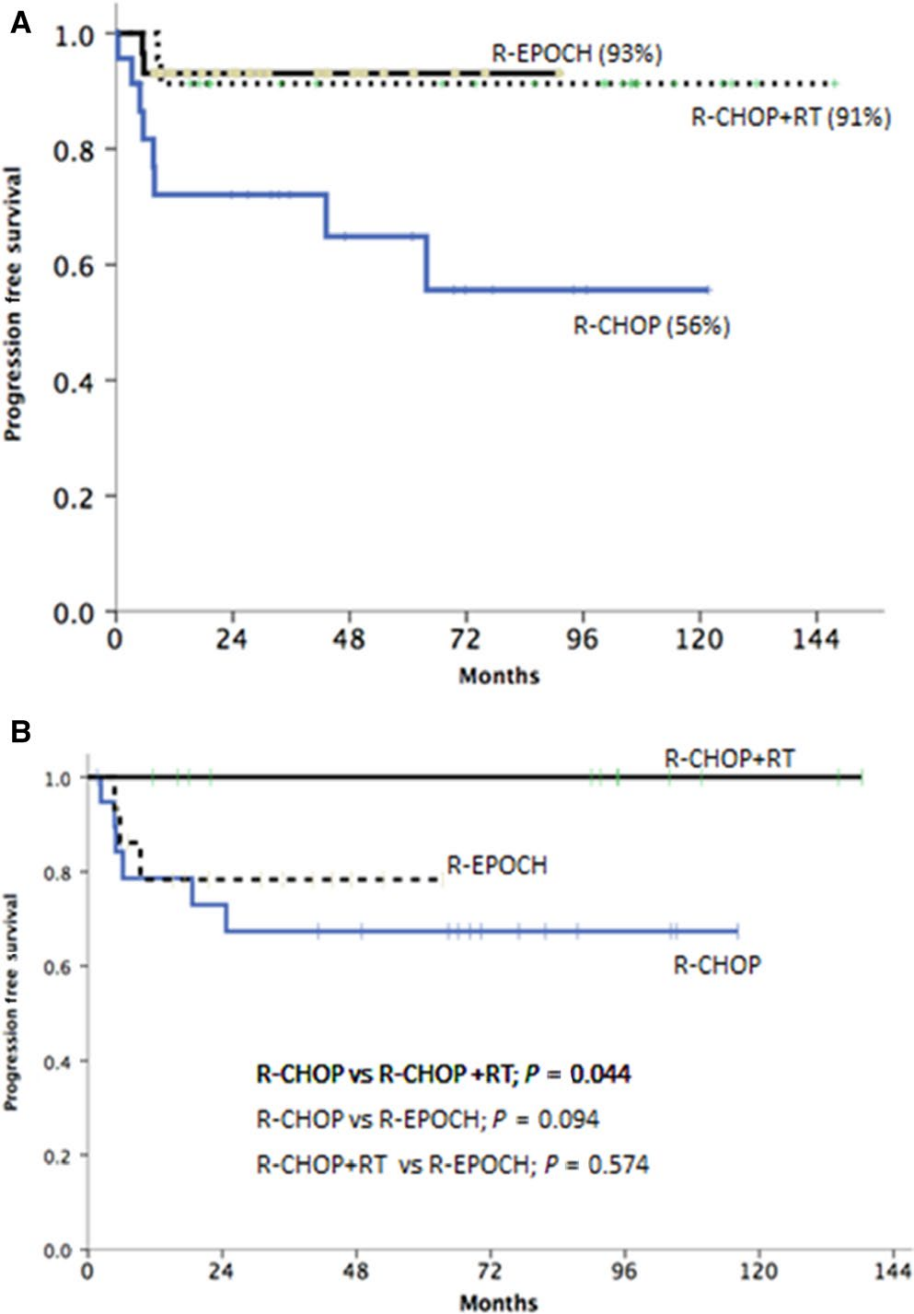
(A) 5 y PFS for Bulky disease subgroup (B) 5 y PFS of non‐bulky disease subgroup

We were also interested to determine if treatment regimen had different effects on PFS in patients with bulky disease vs those without bulky disease. In the subgroup analysis of the patients with bulky disease (n = 71), those who received R‐CHOP alone (n = 21) had an inferior 5‐year PFS as compared to those who received R‐CHOP + RT (n = 23) or DA‐EPOCH‐R (n = 27), with a 5‐year PFS of 56.6% (95% CI, 25.6% to 78.8%), 91.3%(95% CI, 69.5% to 97.8%), and 92.6% (95% CI, 73.5% to 98.1%) respectively (*P* = 0.007) (Figure 3A).

Among the patients without bulky disease, the difference in PFS was not statistically significant (*P* = 0.25) among the three treatment groups, although patients who received R‐CHOP + RT had a trend toward an improved 5‐year PFS as compared to those who received R‐CHOP alone (100% vs 69.3%; *P* = 0.091) but comparable outcome as those who received DA‐EPOCH‐R (100% vs 78.3%; *P* = 0.141) (Figure 3B).

## 4 DISCUSSION

We report a large multicenter cohort of PMBCL patients treated with three treatment regimens widely used in clinical practice. Our study is one of the largest reporting the outcomes in unselected PMBCL patients in the real world setting. Our findings show that both R‐CHOP + RT and DA‐EPOCH‐R are highly effective frontline options for PMBCL. We found that R‐CHOP alone was associated with inferior PFS, especially in patients with bulky disease. While there were more patients with advanced stage disease and higher risk IPI in the R‐CHOP alone group, the treatment regimens retained prognostic significance after multivariate analysis.

Existing retrospective data are conflicting with regards to the value of RT in PMBCL. Our findings are consistent with those reported by Jackson et al where RT was associated with improved outcomes on multivariate analysis. There was however a lack of data on the chemotherapy regimens used in this registry study as compared to our study.^18^ In the UNFOLDER trial (which included 26% of cases with PMBCL), patients with bulky or extra‐nodal DLBCL who achieve CR after R‐CHOP‐based treatment underwent a second randomization to investigate the effect of omitting RT. The two arms without radiation were closed due to treatment failures identified at interim analysis, confirming the importance of RT for patients with DLBCL with bulky disease.^19^ In contrast, population‐based data from British Columbia did not show PFS or OS benefit with the addition of RT in their intention‐to‐treat analysis. However, there were more patients in the chemotherapy alone arm who received more intensive regimens (MACOP‐B/VACOP‐B) compared to CHOP (±R) in the RT arm, due to the era‐specific treatment guidelines used.^20^ It is plausible that the use of more intensive regimens abrogated the benefits of RT.

Our reported outcomes with R‐CHOP + RT are consistent with those reported in two largest prospective studies, the MiNT^6^ and the UK NCRI studies^21^ in their PMBCL subgroup analysis. Both studies recruited patients with localized DLBCL and low risk IPI scores, the majority (especially those with bulky disease > 7.5 cm) of whom received RT consolidation (MinT: 79% and UK NCRI: 58%). The patient characteristics in both studies were very similar to the R‐CHOP + RT population in our study. With a median follow up of 7.2 years, the favorable EFS of 77% and 79.8% seen, respectively, in the PMBCL subsets in these two studies were comparable to that seen in our study.

Our findings provide further evidence to support the use of DA‐EPOCH‐R as an alternative induction regimen, allowing the omission of RT even in patients with bulky disease. Although consolidation RT appears to improve outcomes after R‐CHOP chemotherapy, long‐term toxicity is a serious concern, especially as PMBCL patients are often younger and female. The excellent outcomes of chemotherapy only dose‐intensification with DA‐EPOCH‐R in PMBCL patients is therefore an important consideration in younger patients who are in their twenties to thirties, as it can spare these patients the long term toxicities of mediastinal radiation including increased risk of cardiopulmonary toxicity and death as well as increased breast cancer risk in female patients. Two further multicenter retrospective analysis of frontline DA‐EPOCH‐R in PMBCL have corroborated the excellent outcomes reported by Dunleavy et al^14^ Giulino‐Roth et al studied 156 pediatric and adult patients with PMBCL treated with DA‐EPOCH‐R^22^ The 3‐year EFS and OS were 85.9% and 95.4% respectively. In the second study, Shah et al compared outcomes of R‐CHOP with DA‐EPOCH‐R in a large multicenter retrospective analysis.^16^ Median PFS and OS for both groups were not reached. Although there was a trend for improved hazard ratio in favor of DA‐EPOCH‐R for both PFS and OS, this was not statistically significant. Of note, there were more treatment‐related toxicities in the DA‐EPOCH‐R arm. An important limitation of these two retrospective studies would be selection bias whereby fitter patients were given DA‐EPOCH‐R The authors found no difference in OS between the R‐CHOP alone vs R‐CHOP + RT group. There was, however, a small absolute difference in PFS at 2 years (88% vs 95%) in favor of R‐CHOP + RT, which did not achieve statistical significance, possibly due to the small number of patients in this sub‐analysis. The outcomes in their small (n = 23) R‐CHOP alone subgroup was excellent, with a 2‐year PFS of 88%. This is in contrast with our study which reports an inferior 3‐year PFS of 56.5% for R‐CHOP alone. Importantly, only a minority of DA‐EPOCH‐R treated patients received RT in both studies (13%% in Shah et al and 14.9% in Giulino‐Roth et al). The favorable outcomes of the DA‐EPOCH‐R patients in our study are consistent (5‐year PFS and OS of 88.5% and 94%) with their findings, and only 6% of our patients receiving DA‐EPOCH‐R had RT (Table 2).

**Table 2:** Studies on real world experience with DA‐EPOCH‐R

	Shah et al	Giulino roth et al	Our study (n = 46)
Age (median)	35 (18‐77)	31 (9‐70)	27 (16‐51)
Sex (F)	76 (58%)	100 (64%)	26 (57%)
Stage I‐II (Ann‐arbor)	101 (77%)	84 (73%)	32 (70%)
Bulky disease	92 (78%)	95 (63%)	27 (58%)
IPI 0‐1	81 (66%)	NA	32 (73%)
EFS/PFS	2 y estimate 85%	3 y 86%	5 y 89%
OS	2 y estimate 89%	3 y 95%	5 y 94%

Our study identified B‐symptoms as prognostic for OS and treatment regimen for PFS. Existing non‐randomized and retrospective data are conflicting with regards to the prognostic factors for PMBCL. While some studies have suggested age > 40 years, advanced stage disease and raised LDH as negative prognostic markers for survival, these have not been validated in large prospective studies. In recent years, a Japanese group^6^ developed a PMBCL prognostic score where the presence of a high/intermediate‐risk or high‐risk IPI and the presence of a pleural or pericardial effusion was found to be associated with poorer outcomes amongst the patients treated with R‐CHOP chemotherapy without RT. In our study, apart from impact of treatment group on PFS, we could not identify any other prognostic factor that was significant for PFS on multivariate analysis. Although the Japanese PMBCL prognostic score correlated with PFS on univariate analysis in our study, it did not remain significant on multivariate analysis. These differences with the Japanese data suggest that DA‐EPOCH‐R or the addition of RT to R‐CHOP chemotherapy might overcome the effects of an adverse PMBCL‐IPI.

There are a number of limitations in our study. First, given the retrospective nature of our study, biases in the selection of patients for consolidative RT or choice of chemotherapy is likely. Of note, the population in our study receiving R‐CHOP appeared to have more high‐risk characteristics (with more advanced stage disease and higher IPI) than the R‐CHOP + RT group. Nevertheless, even taking into account these factors, treatment type remained prognostically important on multivariate analysis (especially in the subgroup of patients with bulky disease). In addition, despite the DA‐EPOCH‐R group having more patients with higher risk disease (more advanced disease and higher IPI) compared to the R‐CHOP + RT group, outcomes were comparable in both groups. These findings suggest that the difference in outcomes between the groups were due to treatment modality effect rather than differences in their baseline characteristics alone. Second, most patients did not have imaging performed before RT, and it is plausible that the incorporation of PET would help identify patients with metabolic CR for whom treatment with R‐CHOP alone would have achieved similar results, as suggested in a number of recent retrospective series.^23^ However, findings from the UNFOLDER study suggests that even complete remission on imaging should not be presumed to eliminate the benefit provided by RT, at least in patients with DLBCL.^19^ Therefore, until results from the ongoing IELSG‐37, a Phase 3 randomized trial investigating the role of radiotherapy after a complete metabolic response to rituximab‐containing chemotherapy are available, our findings suggest that RT should be included in patients receiving R‐CHOP, especially for those with bulky disease. Another potential limitation of our study was the lack of central pathology review for the diagnosis of PMBCL; however, all the patients included in our study were treated at tertiary referral centers with experienced hematopathologists. Lastly, our study has limited long term safety data. This is an important consideration, given the need to assess the long‐term complications associated with the addition of etoposide or RT to treatment regimens. Longer follow up would be necessary to address this question. Despite these limitations, our study reflects the real‐world experience of the use of R‐CHOP (±RT) and DA‐EPOCH‐R and has the strengths of a large number of patients, including a large proportion of Asian patients which have not been previously included in other studies. Thus based on our findings, and given the risks of mediastinal toxicity especially in younger patients, we would favor DA R‐EPOCH over R‐CHOP + RT despite the higher costs and practical considerations for a 5‐day infusional regimen.

In conclusion, our findings suggest that both R‐CHOP with consolidation RT and DA‐EPOCH‐R without RT provide excellent outcomes for patients with PMBCL. Additionally, the omission of RT in patients receiving R‐CHOP, especially in those with bulky disease results in inferior PFS. The results of prospective studies in this area to enhance decision making in this important disease are eagerly awaited.
